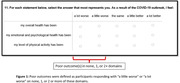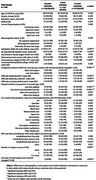# Social, environmental, and cognitive correlates of community‐dwelling older adults coping poorly with reduced social contact: A quasi‐experiment set in Australia’s COVID‐19 lockdown in 2020

**DOI:** 10.1002/alz.092104

**Published:** 2025-01-09

**Authors:** Russell J Chander, Katya T. Numbers, Rory Chen, Suraj Samtani, Nicole A. Kochan, Henry Brodaty, Perminder S. Sachdev

**Affiliations:** ^1^ Centre for Healthy Brain Ageing (CHeBA), University of New South Wales, UNSW Sydney, NSW Australia

## Abstract

**Background:**

The effects of the COVID‐19 pandemic extend beyond the viral impact and include social and psychological effects of the ensuing lockdowns and restrictions. Australia’s lengthy lockdowns present an opportunity to study changes in the physical and mental wellbeing of older adults resulting from extended social isolation, a known risk factor for dementia, in the absence of high infection or mortality rates.

**Method:**

Sydney Memory and Ageing Study, Sydney Centenarian Study, and CogSCAN study participants were mailed questionnaires about in‐person and remote social contact and access to resources during the 2020 Sydney lockdown. Responses were linked to existing medical history and cognitive status data from 2018‐2019. Respondents were classified based on self‐reported poor outcomes (PO), defined as feeling worse because of the pandemic in three domains: overall health, emotional and psychological health, and physical activity. Cognitive impairment was defined as presence of DSM‐V neurocognitive disorders as determined by clinical consensus, MMSE <27, or ACE‐III <88. Factors associated with PO were explored.

**Result:**

Of the 335 participants who responded to the COVID‐19 questionnaire by January 2021, 75 (22.4%) met criteria for cognitive impairment. None of the respondents reported ever being symptomatic at the time of study. Only 19 (5.7%) personally knew someone who had COVID‐19. Most (156, 46.7%) participants had no POs, 112 had one, and 67 had two or more. Cognitive status was not associated with POs. Compared to no POs, PO participants actively reduced in‐person contact, overall social contact, had more difficulty accessing food and medical resources. Despite PO participants communicating more frequently with friends and family by phone, and reporting having changed their social support, they also had lower scores in questionnaires for life satisfaction and loneliness.

**Conclusion:**

Older adults who reported poorer outcomes during lockdown had greater reduced social contact. Despite efforts to adapt their social support networks through increased phone‐based social interactions, they still reported worse life satisfaction and more loneliness. Difficulties in adapting to changing social circumstances and isolation may confer a greater susceptibility to loneliness. Further work is needed to understand the relationship between susceptibility to loneliness and future dementia risk.